# Rhizosphere Microbiomes Modulated by Pre-crops Assisted Plants in Defense Against Plant-Parasitic Nematodes

**DOI:** 10.3389/fmicb.2018.01133

**Published:** 2018-06-04

**Authors:** Ahmed Elhady, Shimaa Adss, Johannes Hallmann, Holger Heuer

**Affiliations:** ^1^Department of Epidemiology and Pathogen Diagnostics, Julius Kühn-Institut – Federal Research Centre for Cultivated Plants, Braunschweig, Germany; ^2^Department of Plant Protection, Faculty of Agriculture, Benha University, Benha, Egypt

**Keywords:** phytobiome, rhizosphere, microbiome, induced resistance, plant-parasitic nematodes

## Abstract

Plant-parasitic nematodes cause considerable damage to crop plants. The rhizosphere microbiome can affect invasion and reproductive success of plant-parasitic nematodes, thus affecting plant damage. In this study, we investigated how the transplanted rhizosphere microbiome from different crops affect plant-parasitic nematodes on soybean or tomato, and whether the plant’s own microbiome from the rhizosphere protects it better than the microbiome from fallow soil. Soybean plants growing in sterilized substrate were inoculated with the microbiome extracted from the rhizosphere of soybean, maize, or tomato. Controls were inoculated with extracts from bulk soil, or not inoculated. After the microbiome was established, the root lesion nematode *Pratylenchus penetrans* was added. Root invasion of *P. penetrans* was significantly reduced on soybean plants inoculated with the microbiome from maize or soybean compared to tomato or bulk soil, or the uninoculated control. In the analogous experiment with tomato plants inoculated with either *P. penetrans* or the root knot nematode *Meloidogyne incognita*, the rhizosphere microbiomes of maize and tomato reduced root invasion by *P. penetrans* and *M. incognita* compared to microbiomes from soybean or bulk soil. Reproduction of *M. incognita* on tomato followed the same trend, and it was best suppressed by the tomato rhizosphere microbiome. In split-root experiments with soybean and tomato plants, a systemic effect of the inoculated rhizosphere microbiomes on root invasion of *P. penetrans* was shown. Furthermore, some transplanted microbiomes slightly enhanced plant growth compared to uninoculated plants. The microbiomes from maize rhizosphere and bulk soil increased the fresh weights of roots and shoots of soybean plants, and microbiomes from soybean rhizosphere and bulk soil increased the fresh weights of roots and shoots of tomato plants. Nematode invasion did not affect plant growth in these short-term experiments. In conclusion, this study highlights the importance of the rhizosphere microbiome in protecting crops against plant-parasitic nematodes. An effect of pre-crops on the rhizosphere microbiome might be harnessed to enhance the resistance of crops towards plant-parasitic nematodes. However, nematode-suppressive effects of a particular microbiome may not necessarily coincide with improvement of plant growth in the absence of plant-parasitic nematodes.

## Introduction

Cultivated plants in agroecosystems are part of multi-organismal associations called phytobiomes that may support them in nutrient acquisition, production of growth factors, and defense against pathogens depending on its composition (Sánchez-Cañizares et al., 2017). Plant-parasitic nematodes are microscopic worms that migrate through soil in search of a host plant where they feed from the liquid content of root cells. Root damage in association with the withdrawal of plant nutrients finally leads to plant damage and yield losses. Globally, crop losses associated with plant-parasitic nematodes are estimated to be 12.6% corresponding to $216 billion per year (Nyaku et al., 2017). However, damage by plant-parasitic nematodes often remains unnoticed as aboveground symptoms are rare and a proper diagnosis for nematodes is lacking. Plant-parasitic nematodes vary in their life cycle and type of parasitism. The root lesion nematode *Pratylenchus penetrans* is an endoparasite that invades and migrates through roots as juvenile or adult without becoming sedentary, and escapes to soil under adverse conditions inside roots. In contrast, the root-knot nematode *Meloidogyne incognita* is a sedentary endoparasite, where the infectious second-stage juveniles (J2) enter the root near the tip and establish a feeding site near the vascular system. Once done, the juvenile loses its motility and completes its life cycle within the root.

Management of plant-parasitic nematodes is a challenge because nematicides have been mostly banned, and resistant varieties or non-host crops are often not available or not profitable. Novel methods with high potential and sustainability have become an urgent need. Biocontrol agents are quite expensive and often do not provide consistent control. Interestingly, there is mounting evidence that the indigenous plant microbiome plays a vital role in suppressing soil-borne diseases (Besset-Manzoni et al., 2018). Soil microbiomes that suppressed root invasion and reproduction of plant-parasitic nematodes have been described (Bent et al., 2008; Adam et al., 2014; Elhady et al., 2017). It has been suggested to harness synthetic consortia of microbes (Julien-Laferrière et al., 2016), or beneficial microbiomes (Nyaku et al., 2017) to increase the sustainability and productivity of agriculture. To avoid expensive and inconsistently efficient inoculation of plants with microbes for improvement of plant growth and health, engineering of the soil microbiome was suggested (Finkel et al., 2017). Naturally, the plants itself modulate the microbiome in the rhizosphere (Venturi and Keel, 2016). This may be effective for a prolonged period of time and affect the next plant growing in the same soil (Lapsansky et al., 2016). Agronomically, a preceding crop can affect the yield of a following crop (Jacobs et al., 2018). Understanding plant-microbiome feedbacks was suggested to be one of the keys for exploiting the yield potential of cropping systems (Ahkami et al., 2017). It is yet unclear how different crops affect each other by their specific microbiomes. In monoculture systems soils eventually become disease-suppressive to plant-parasitic nematodes over time (Hamid et al., 2017). However, it was not investigated whether plants enrich a microbiome in their rhizosphere that is more beneficial to them than the microbiome from bulk soil or a different pre-crop.

In this study, we investigated how the rhizosphere microbiomes of different plants affect root invasion of plant-parasitic nematodes on soybean and tomato, and whether the plant’s own microbiome protects it better than the microbiome from bulk soil or a different pre-crop. It was analyzed in split-root systems whether observed effects of the microbiome on the nematodes were plant-mediated, and whether induced resistance against the plant-parasitic nematodes has a trade-off in plant growth.

## Materials and Methods

### Plants, Growth Conditions, and Microbiome Inocula

In order to obtain the soil and rhizosphere microbiome inocula, soybean (*Glycine* *max*) cv. Primus; maize (*Zea mays*) cv. Colisee and tomato (*Solanum lycopersicum*) cv. Moneymaker were grown in 12 cm diameter plastic pots filled with 500 ml field soil as donor crop. The soil was a loamy sand, braunerde, pH 6.5, from a field in Braunschweig, Germany (52°17^′^57^′′^ N, 10°26^′^14^′′^ E). Cultivation of soybean in the previous year (tillage, NPK fertilization) resulted in a density of *Pratylenchus* of 599 ± 11 infective stages per 100 ml soil, while other plant-parasitic nematodes had low adundances (*Paratylenchus* 76 ± 29 per 100 ml, *Tylenchorhynchus* 140 ± 21 per 100 ml). The field soil was sampled in May before planting and kept at 4°C until preparation of rhizosphere and bulk soil microbiomes. Five plants per pot were grown to guarantee sufficient root biomass and raise the potential of modulating the tested soil by the donor crops. Pots with only field soil were left fallow to provide the microbiome of bulk soil serving as non-modulated control. All pots were watered as needed every 2–3 days and fertilized weekly with 5 ml per 100 ml substrate with commercial fertilizer (WUXAL Super NPK fertilizer, 8-8-6 with micronutrients, 2.5 g/l, AGLUKON, Düsseldorf, Germany). Pots were kept in the greenhouse at 24°C and 16 h photoperiod. Two weeks after the donor crops were planted, soybean and tomato was planted as recipient hosts for the rhizosphere and bulk soil microbiomes. The soybean and tomato seeds were surface sterilized with 1.5% sodium hypochlorite for 15 min and then rinsed 5 times with sterile deionized water. The seeds were germinated for 5 days on paper tissue under sterile conditions. Seedlings were then planted into two times autoclaved sand as an artificial growth substrate, i.e. in pots containing 100 ml sand for the nematode penetration assay and in pots containing 500 ml sand for the reproduction assay. Recipient plants were grown for 10 days before inoculation of a microbiome suspension from donor crops or bulk soil. For that, the microbiomes of roots with 15 g rhizosphere soil of 6 week-old donor crops, or 15 g bulk soil were extracted in a Stomacher blender (Seward, London, United Kingdom) three times with 15 ml sterile 0.85% NaCl at high speed for 60 s. Soil particles were sedimented and the microbial suspensions of the supernatant were passed through a 5 μm sieve to remove remaining particles, nematodes, and root debris. The microbes were pelleted for 10 min at 4000 g and re-suspended in 45 ml sterile tap water. Each pot with the recipient plant received 15 ml of this suspension per 100 ml autoclaved sand. The transplanted microbiomes were established for 2 weeks in the rhizosphere of the recipient plants before plant-parasitic nematodes were inoculated.

### Growth and Surface Sterilization of Plant-Parasitic Nematodes

Adults and juveniles of *P. penetrans* were multiplied for 2–4 months on carrot disks and extracted by Baermann funnel (EPPO, 2013). The root knot nematode *M. incognita* was multiplied on tomato cv. Moneymaker for 2 months in the greenhouse at 16 h photoperiod and 26°C. Second-stage juveniles (J2) were collected by picking egg masses from tomato roots and transferring them into sterile tap water at room temperature to facilitate hatch of J2. For surface disinfection, nematodes were placed first on 5 μm sieves (Cell-Trics1 filters, Sysmex, Norderstedt, Germany) and washed with 10 ml sterilized tap water. Nematodes were then treated with 0.02% HgCl_2_ for 3 min and with 4000 ppm streptomycin sulfate for another 3 min. Next, nematodes were incubated for 4 h in 5 ml 1x CellCultureGuard (AppliChem, Darmstadt, Germany) on a rotary shaker at 150 rpm. Finally, the nematodes were washed on a 5 μm sieve and incubated overnight in sterilized tap water. Prior to use in the experiments, nematodes were checked for their sterility by plating them on R2A (Merck, Darmstadt, Germany) for bacterial growth and on potato extract glucose agar (Carl Roth, Karlsruhe, Germany) for fungal growth. Inoculation of plants with nematodes was done by digging eight ca. 5 cm deep half an inch wide holes in 5 cm distance around the shoot, and equally distributing the nematode suspension.

### Nematode Invasion and Reproduction Assays

Ten days after nematode inoculation, roots were sampled and washed to remove soil. Nematode invasion was quantified by staining the roots with 1% acid fuchsin (Bybd et al., 1983). Roots were stored in the staining solution at 4°C until counting of nematodes at 20× magnification under a stereomicroscope. To determine the number of living and dead nematodes in the growth substrate, 250 g soil was washed over a sieve combination of 100 μm and 5 μm. The soil particles on the 100 μm sieve were discarded and the nematodes on the 5 μm sieve counted under a stereomicroscope. Alive and dead nematodes were distinguished based on their active mobility and body style.

To determine the reproduction of *M. incognita*, roots were sampled 2 months after nematode inoculation. Roots were gently washed to clean them from adhering soil. The number of galls on the root was determined. To estimate the number of eggs, roots were cut to 2 cm pieces and macerated in 1.5% sodium hypochlorite twice for 15 s each with a commercial blender (Waring, Torrington, CT, United States). The macerate was passed through a 100 μm sieve nested over a 5 μm sieve. Plant debris collected on the 100 μm sieve was discarded and eggs collected on the 5 μm sieve were washed with tap water into 50 ml falcon tubes. A 1 ml aliquot was transferred into a nematode-counting slide and nematodes were counted at 40× magnification under a stereomicroscope.

### Invasion of *P. penetrans* Into Soybean Roots Affected by Transplanted Microbiomes

In order to investigate how the transplanted rhizosphere microbiome from different crops affect invasion of *P. penetrans* into roots, soybean plants were grown in two times steam-sterilized sand. Two weeks old soybean plants were inoculated with microbiomes from soybean, tomato, or maize rhizosphere, or from bulk soil in randomized complete block design with 12 replicates. Plants were kept for 2 weeks for microbiome establishment and colonization before each pot was inoculated with 1000 mixed stages of *P. penetrans.* The number of *P. penetrans* in roots, and the living and dead *P. penetrans* in soil were determined 10 days after inoculation.

### Susceptibility of Tomato to *P. penetrans* and *M. incognita* Affected by Transplanted Microbiomes

An analogous experiment with tomato was used to confirm whether the plant’s own microbiome protects it better than the microbiome from bulk soil or a different pre-crop. Each pot was inoculated with 500 mixed stages of *P. penetrans* or J2 of *M. incognita*. The invasion of both nematodes was determined after 10 days while the reproduction of *M. incognita* and their galls was determined 2 months after incubation. Each treatment had 10 replicates.

### Split-Root Experiment

The potential of the rhizosphere microbiome to induce systemic resistance was studied in a split-root system as described by Dababat and Sikora (2007). Three square pots of 7 cm × 7 cm × 8 cm were arranged as follows: Two pots were attached to each other (inducer pot and responder pot, respectively) and one pot was placed in the center above those two pots. Two-week old seedlings of tomato or soybean were transplanted in the center of the upper pot, which was half filled with sterile sand. The number of replicates was 10 for tomato and 12 for soybean. The inducer pot was inoculated with the microbiomes of the donor crops. After establishment of the microbiome, the responder pot was inoculated with 500 mixed stages of *P. penetrans*. The plants were watered and fertilized. The roots were weighted and the number of invaded *P. penetrans* was determined. Roots of the inducer side were frozen in liquid nitrogen for later determination of phenolic compounds. The total phenolic compounds in 0.5 g root were quantified using a Folin Ciocalteu assay (Ainsworth and Gillespie, 2007), with gallic acid (Sigma-Aldrich, Darmstadt, Germany) as reference for quantification.

### Statistical Analysis

Analysis of variance was done using the procedure GENMOD of the statistical software SAS 9.4 (SAS Institute Inc., Cary, NC, United States) to fit generalized linear models. For count data (numbers of galls, eggs, nematodes) the procedure was used to perform a Poisson regression analysis with a log link function and specification of a scale parameter (Pearson) to fit overdispersed distributions. Class variables were treatment (microbiome or uninoculated control) and block (accounting for the randomized block design of experiments). For multiple comparisons, the *p*-value was adjusted by the method of Tukey. Graphs were generated using Prism 7 (GraphPad Software, La Jolla, CA, United States).

## Results

### Rhizosphere Microbiomes of Different Crops Affected the Invasion of *P. penetrans* Into Soybean Roots

**FIGURE 1:**
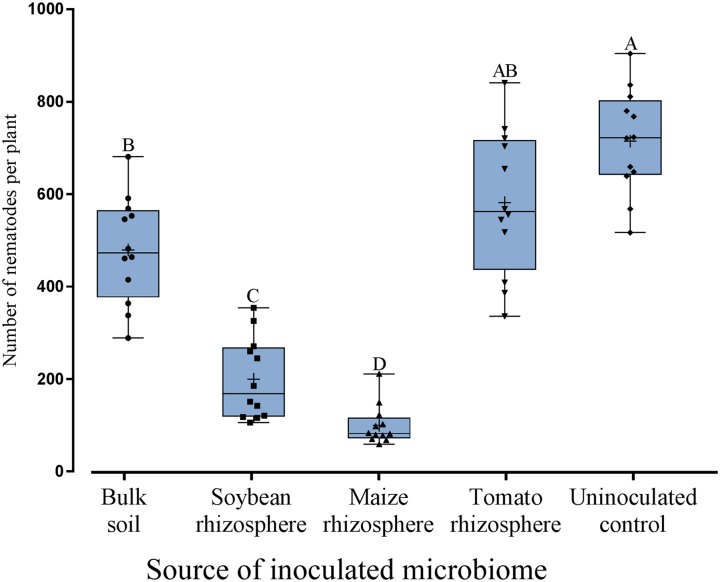
Effect of different microbiomes transplanted from the rhizosphere of different donor crops or bulk soil to the rhizosphere of soybean on the invasion of *Pratylenchus penetrans* into roots. Mean numbers of *P. penetrans* in the root 10 days after inoculation of the nematodes into soil are shown as (+) for each treatment, the medians are shown as (—), whiskers indicate quartiles. Different letters above whiskers indicate significant differences among treatments in Tukey’s test (*n* = 12).

**FIGURE 2:**
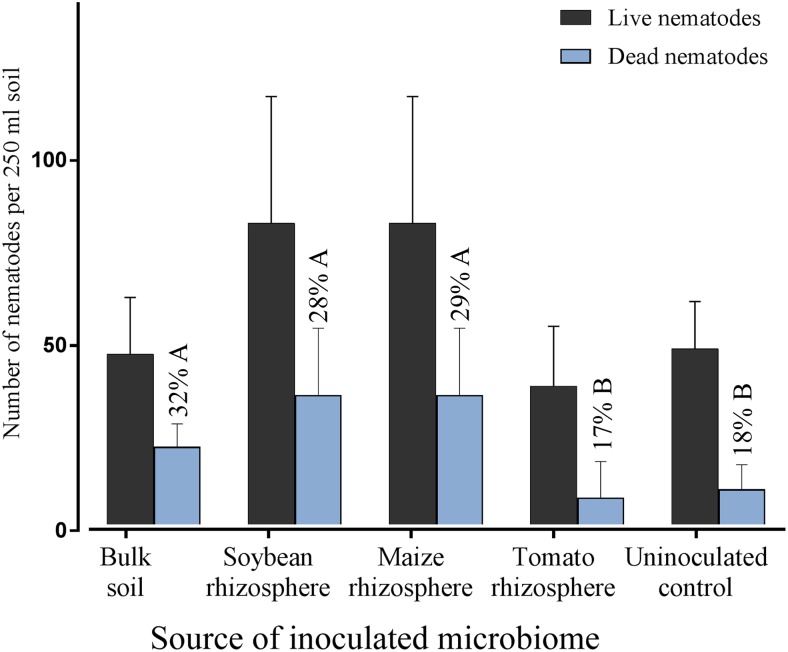
Densities of living and dead *Pratylenchus penetrans* in the soil fraction of pots with soybean plants that were inoculated with microbiomes from different sources, or not inoculated with microbes, 10 days after inoculation of the nematodes. Percentages of dead *P. penetrans* in each treatment are shown above the blue bars, different letters indicate significant differences in Tukey’s test (*n* = 12). Error bars represent standard deviations.

Microbes extracted from the rhizosphere of soybean, maize, tomato and from bulk soil were inoculated to the roots of soybean to investigate their effect on root invasion by *P. penetrans*. The type of inoculated microbiome significantly affected the number of nematodes that penetrated the root (**Figure 1**). Compared to the sterile control the microbiomes from soybean, maize and bulk soil all significantly reduced the invasion of *P. penetrans* (**Figure 1**). The tomato microbiome showed a similar trend, although not significantly. The inoculated microbiomes from soybean and maize rhizospheres affected the nematodes stronger than the microbiome from bulk soil, while the effect of the tomato microbiome did not significantly differ from that of bulk soil. The strongest effect on *P. penetrans* was exerted by the microbiome of the maize rhizosphere that reduced invasion significantly more than the microbiome from the soybean rhizosphere. Regarding dead and alive specimen of *P. penetrans* outside the root, dead nematodes were relatively higher in the substrates treated with the microbiomes of bulk soil, maize rhizosphere and soybean rhizosphere than in the uninoculated control, suggesting a role of direct antagonism of the microbiome to the nematode (**Figure 2**). For the microbiome of the tomato rhizosphere such an effect was not observed. The numbers of live *P. penetrans* in the substrate were significantly higher in the pots with microbiomes from maize and soybean compared to all other treatments. Thus, the lower invasion rates of these treatments could not be explained by microbe-induced death of *P. penetrans* outside the root but rather by preferential partitioning of the active nematodes.

### Effect of Different Rhizosphere Microbiomes on Invasion of *P. penetrans* and *M. incognita* Into Tomato Roots

**FIGURE 3:**
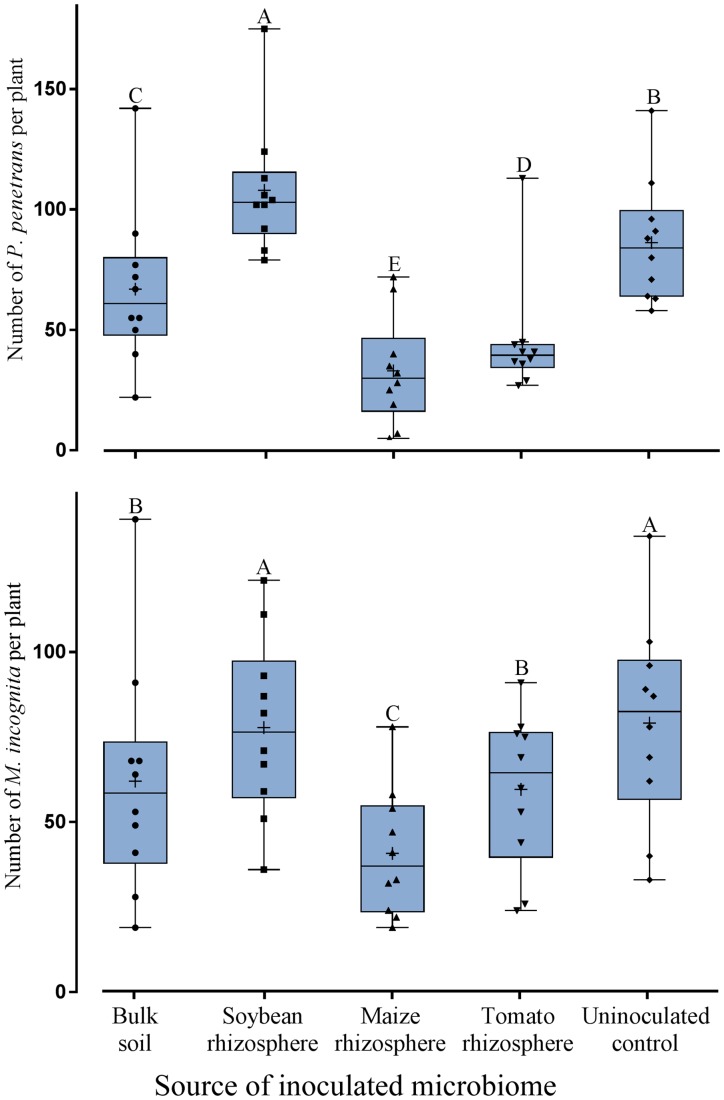
Effect of transplanted microbiomes from the rhizosphere of different donor crops or bulk soil on the number of *Pratylenchus penetrans* (migratory endoparasitic nematodes) or *Meloidogyne incognita* (sedentary endoparasitic nematodes) in tomato roots 10 days after inoculation of the nematodes into soil. Mean numbers are shown as (+) for each treatment, medians are shown as (—), whiskers represent quartiles. Different letters indicate significant differences among treatments in Tukey’s test (*n* = 10).

To investigate whether the defense supportive effect of a plant’s own microbiome in the rhizosphere is specific to soybean, tomato plants were used as a microbiome recipient instead of soybean. The effect of transplanted microbiomes on tomato root invasion was analyzed for two species of plant-parasitic nematodes that differ in their parasitism and life cycles, i.e., the migratory endoparasite *P. penetrans* and the sedentary endoparasite *M. incognita*. The invasion of tomato roots by both nematodes significantly depended on the inoculated microbiome (**Figure 3**). The trend of the interactions with the different microbiomes was similar for both nematodes. All inoculated microbiomes except from soybean significantly reduced the invasion of *P. penetrans* and *M. incognita* into tomato roots compared to the uninoculated control (**Figure 3**). The soybean rhizosphere microbiome failed to hamper both *P. penetrans* and *M. incognita* to invade into tomato roots. The microbiomes from maize and tomato rhizospheres significantly reduced the invasion of *P. penetrans* into the tomato roots compared to the bulk soil microbiome. For *M. incognita* only the treatment with maize microbiome and not tomato microbiome differed from the effect of the bulk soil microbiome on root invasion of the nematode. The microbiome extracted from maize rhizosphere had the highest suppressive effect among the tested microbiomes against both *P. penetrans* and *M. incognita.* Comparison with the previous experiment showed that soybean plants are better protected from invasion of the nematodes by the soybean microbiome rather than the tomato microbiome, and that tomato plants are better protected by the tomato microbiome rather than the soybean microbiome.

**FIGURE 4:**
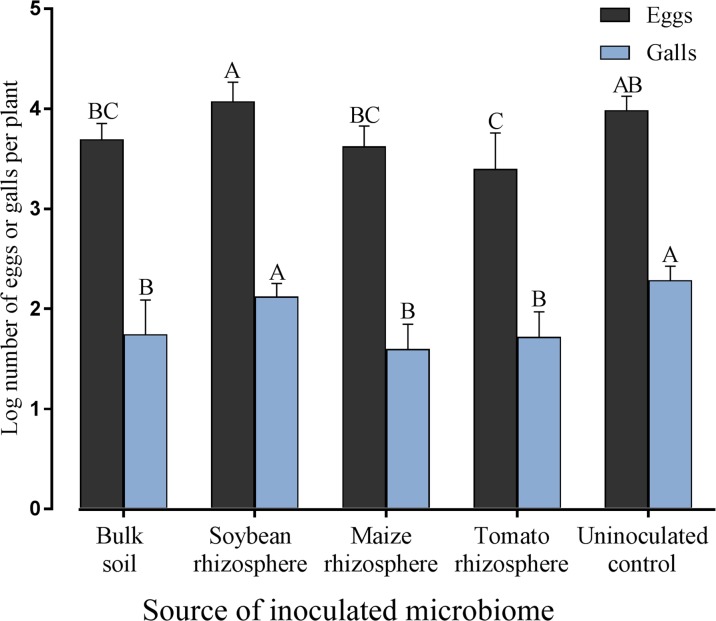
Effect of transplanted microbiomes from the rhizosphere of different donor crops or bulk soil on galls and eggs produced by *Meloidogyne incognita* on tomato plants two months after inoculation of the nematodes. Different letters indicate significant differences among treatments in Tukey’s test (*n* = 10). Error bars represent standard deviations.

To investigate the effect of different rhizosphere microbiomes of tomato plants on the reproduction of *M. incognita*, the number of galls and eggs were determined 2 months after nematode inoculation of tomato plants previously inoculated with the microbiome from soybean rhizosphere, maize rhizosphere, tomato rhizosphere, bulk soil, or uninoculated. The number of galls on tomato roots was significantly reduced in the treatments with transplanted microbiomes compared to the control, except for the microbiome from soybean rhizosphere (**Figure 4**). The number of eggs was only on those tomato plants significantly reduced which received the rhizosphere microbiome from tomato plants (**Figure 4**). On tomato plants that received the microbiome from soybean plants the largest offspring was produced by *M. incognita*.

### Plant-Mediated Effect of Microbiomes on *P. penetrans* Invasion Into Soybean and Tomato Roots Analyzed in Split-Root Systems

**FIGURE 5:**
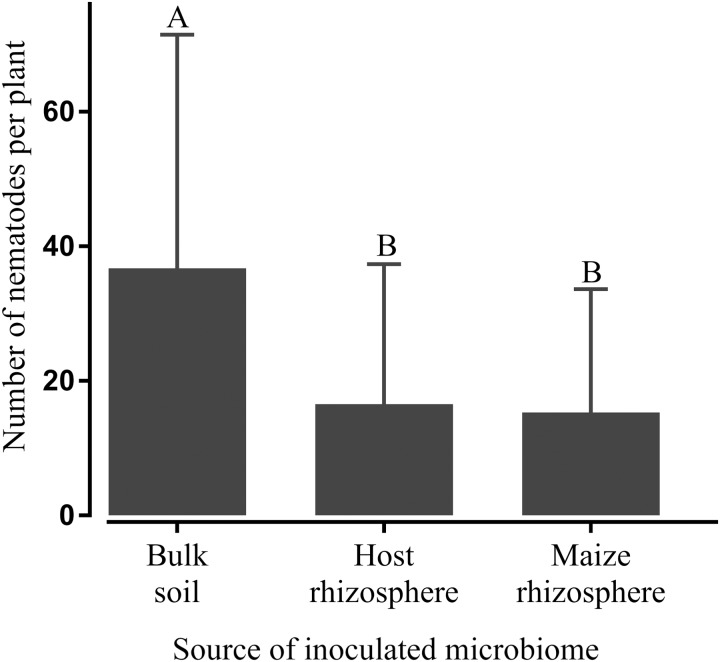
Systemic effect of microbiomes transplanted from the rhizosphere of the host plant (tomato or soybean) or maize, or bulk soil on the number of *Pratylenchus penetrans* in the root of the host plant 10 days after inoculation of the nematodes. Microbiomes were inoculated on one side of a split-root system and nematodes on the opposite side. Different letters indicate significant differences among treatments in Tukey’s test (*n* = 10). Error bars represent standard deviations.

To investigate whether the observed effect of some microbiomes on nematode invasion of roots is plant-mediated or rather based on a direct antagonism of microbes towards the nematodes, microbes and *P. penetrans* were inoculated spatially separated in split root systems of tomato and soybean plants. Again, the microbiomes extracted from the rhizospheres of plants of the receiving crop or maize significantly reduced the number of invaded *P. penetrans* in the roots compared to the treatment with the microbiome from bulk soil (**Figure 5**). This suggested an involvement of systemic resistance of the plant specifically induced by these microbiomes as a basis for the observed effects. The invasion rates of nematodes into the roots did not differ between the treatments with host and maize microbiomes. Soybean and tomato plants showed the same trend to stronger suppress root invasion by nematodes when inoculated with the rhizosphere microbiome from the same plant species compared to inoculation with the bulk soil microbiome.

### Effect of Rhizosphere Microbiomes on Plant Growth

**FIGURE 6:**
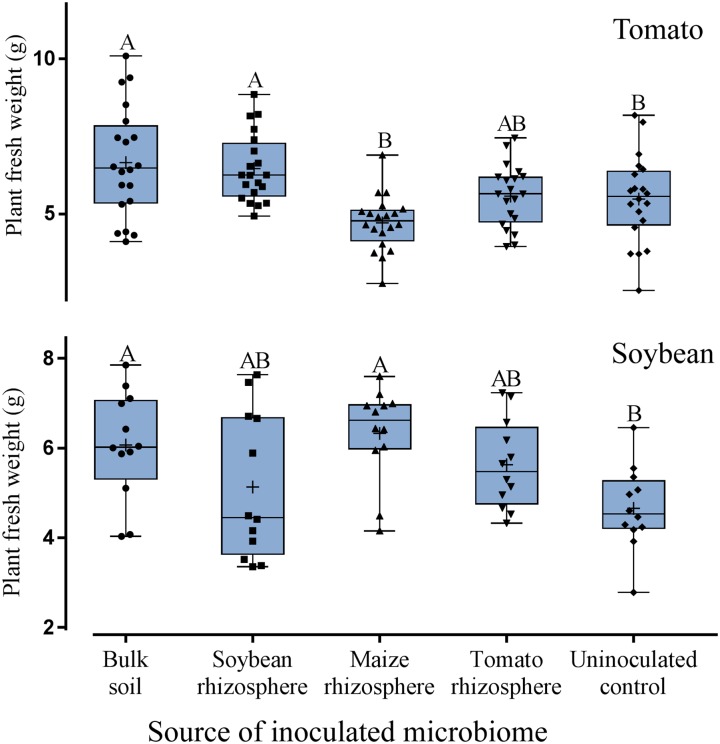
Effect of microbiomes transplanted from the rhizospheres of different donor crops or bulk soil on the weight of tomato plants (upper panel, *n* = 10) or soybean plants (lower panel, *n* = 12). Mean numbers are shown as (+) for each treatment, medians are shown as (—), whiskers represent quartiles. Different letters indicate significant differences among treatments in Tukey’s test.

Although treatment effects on plant growth could not be expected due to the short experimental period, a trend for higher plant weight was observed for tomato and soybean plants inoculated with microbiomes compared to the uninoculated control (**Figure 6**). A slightly better growth than in the uninoculated control was determined for tomato with microbiomes from bulk soil or from soybean rhizosphere, and for soybean with microbiomes from bulk soil or maize rhizosphere (**Figure 6**). Overall, by applying a generalized linear model, the type of microbiome had a statistically significant effect on plant weight but this effect was rather weak when looking at the root and shoot weights of the single experiments (**Supplementary Table S1**). Numbers of leaves per plant showed no significant differences among all the treatments. Notably, the microbiome from maize rhizosphere supported the least growth of tomato plants while it well protected tomato plants from nematodes, suggesting a trade-off between growth and defense. The plant weight was not negatively influenced by the number of nematodes in the root but showed rather a slight positive correlation.

In the split-root experiment, the root weight of tomato was significantly increased on the side of the inoculated microbiomes compared to the side without inoculated microbiome (**Table 1**). The tomato microbiome supported root growth of tomato significantly better than microbiomes from maize rhizosphere or bulk soil on the inoculated side of the root system, while no significant difference was observed on the other side, which was inoculated with nematodes instead of microbiome. For soybean root weight determined on the nematode side, no significant difference was found between treatments. To investigate whether the microbiomes induced stress responses of the root that might affect growth of the plant, the concentration of total phenolic compounds in the roots on the microbiome side was determined. The type of the inoculated microbiome and the recipient plant had a statistically significant effect on the concentration of total phenolics (**Table 1**). However, the means differed by maximally 7%. In tomato roots, the total phenolics were increased by its own microbiome compared to bulk soil and maize microbiomes. In soybean, the maize microbiome increased the accumulated phenolics more than other microbiomes. Overall, the rhizosphere microbiomes showed a trend for higher induction of phenolic compounds production than the bulk soil microbiome (**Table 1**).

**Table 1:** Total phenolics and root fresh weight of soybean and tomato inoculated with different microbiomes from the rhizosphere of different donor crops or bulk soil, and uninoculated control (different letters in a column indicate significant differences in Tukey’s test).

Source of inoculated microbiome	Tomato plants (*n* = 10)			Soybean plants (*n* = 12)	
	Total phenolics^1^	Root fresh weight (g)		Total phenolics^1^	Root fresh weight (g) in nematodes side
		Microbiome side	Nematodes side		
Bulk soil	1.63 ± 0.09b	0.70 ± 0.23b	0.49 ± 0.25a	1.80 ± 0.12b	0.54 ± 0.23a
Host rhizosphere	1.75 ± 0.10a	1.59 ± 0.62a	0.84 ± 0.49a	1.85 ± 0.08b	0.66 ± 0.15a
Maize rhizosphere	1.64 ± 0.05b	0.97 ± 0.24b	0.64 ± 0.27a	1.94 ± 0.07a	0.69 ± 0.15a

*^1^In roots on the microbiome side (mg gallic acid equivalent per gram root)*.

## Discussion

In this study, rhizosphere microbiomes of different plants affected the invasion of the plant-parasitic nematodes *P. penetrans* and *M. incognita* into roots of soybean and tomato plants, and reduced reproduction of *M. incognita* in tomato roots. This suppressive effect depended on the plant species from which the microbiome was transplanted. Most efficient in suppression of the nematodes on both plants was the microbiome enriched in the rhizosphere of maize. We tested the effect of the different microbiomes in a standardized pot system containing sterile substrate to avoid confounding factors of physico-chemical soil properties. Indigenous plant-parasitic nematodes were removed by sieving because inter-species competition might affect root invasion and reproduction assays (Umesh et al., 1994). Some evidences of an effect of soil microbiota on root-knot nematodes were reported in earlier studies (Pyrowolakis et al., 2002; McSorley et al., 2006). Adam et al. (2014) found significantly lower reproduction of *Meloidogyne hapla* on tomato plants growing in native soils compared to disinfected soils which showed the importance of soil microbiota in the process. The suppressive effect of the soil microbiota differed between soils from several fields with different crop rotations, and a soil with maize as pre-crop was most suppressive. However, it remained unclear in how far the effect was due to an influence of the pre-crop on the microbiome. In addition, a bias by differing abiotic properties of the soils or native plant-parasitic nematodes could not be ruled out. In our study, the suppressive effect of rhizosphere microbiota was more pronounced than that of bulk soil microbiota. This might be explained by the composition of the microbiome in the plant rhizosphere but also by the higher density of microbes with a higher metabolic activity compared to the microbiome of the bulk soil (Philippot et al., 2013).

For tomato and soybean plants we showed that the plant’s own microbiome protected it better from plant-parasitic nematodes than the microbiomes from bulk soil or from the respective other plant. Each plant species recruits a specific set of root associated microbes when planted in the same soil (Turner et al., 2013; Haichar et al., 2014; Ofek et al., 2014). The plant’s own specific rhizosphere microbiome might have coevolved with the plant to assist in growth and defense (Sánchez-Cañizares et al., 2017). Enrichment of the plant’s own microbiome in mono-cropping soils might explain the often observed development of suppressiveness to specific pathogens of that crop (Zhu et al., 2013; Hamid et al., 2017). However, in our study not the plant’s own microbiome but the microbiome from the maize rhizosphere had the most suppressive effect against *P. penetrans* and *M. incognita* on soybean and tomato plants. This leads to the conclusion that maize might be a good pre-crop in rotations with soybean and tomato with respect to managing the soil microbiome. The rhizosphere microbiome of maize was shown to harbor a higher functional diversity than bulk soil (Li et al., 2014a,b) and is enriched with bacterial taxa of the orders Burkholderiales, Oceanospirillales, Sphingobacteriales, Actinobacteria, and Bacteroidetes containing several beneficial species (Chauhan et al., 2011; Peiffer et al., 2013). Furthermore, it was shown that root exudates of maize can stimulate rhizosphere colonization by *Bacillus amyloliquefaciens* SQR9 resulting in enhanced plant growth and reduced infestation by soil pathogens (Zhang et al., 2015). In addition, secondary metabolites of maize like benzoxazinoid induce plant defense mechanisms against soil pathogens and contribute to the recruitment of plant beneficial bacteria in the maize rhizosphere (Neal et al., 2012).

Plant-parasitic nematodes migrate through the soil in search of roots, directed by communication signaling, and thereby interfere with indigenous microbes. This could be a direct antagonism, or microbes in the rhizosphere can stimulate plant defenses and thus interfere with plant-parasitic nematodes indirectly. In this study, we observed that the suppressive effect of the microbiome on *P. penetrans* was at least partially mediated by the plant as shown in the split-root experiment. This was also evidenced by the observation that *P. penetrans* partitioned more into the compartment outside the root in the pots with suppressive microbiomes, and less into the root, compared to less suppressive treatments. At the same time the death rate of *P. penetrans* in soil did not contribute to suppressiveness. The plant-mediated effect of the rhizosphere microbiomes could be caused by stimulation of the biosynthesis of phytohormones, defense proteins and secondary metabolites that are involved in plant defense responses (Pieterse et al., 2014; Rashid and Chung, 2017). A major role in the regulation of plant defenses play phenolic compounds (Ma et al., 2016). Our results showed that the type of the inoculated microbiome significantly affected accumulation of total phenolics in the roots, i.e., accumulation of phenolic compounds was highest in soybean roots treated with the rhizosphere microbiome of maize and in tomato roots treated with the microbiome of tomato. In a recent study, the microbiome of a suppressive soil increased resistance of tomato plants against *Fusarium oxysporum* f. sp. *lycopersici* compared to steam disinfected soil by inducing a state of alert which included increased levels of phenolic compounds in the roots (Chialva et al., 2018).

In our study, the plant-mediated effect on the parasitic nematodes depended on the plant species from which the microbiome was transplanted. This effect might be harnessed to engineer soil microbiomes by selected crops towards increased plant resistance against plant-parasitic nematodes (Nyaku et al., 2017). However, a trade-off between induced resistance and plant growth was often reported (Huot et al., 2014; Vyska et al., 2016). With the exception of tomato plants treated with the rhizosphere microbiome of maize, plants inoculated with microbiomes showed a higher plant fresh weight than non-inoculated plants. On the contrary, the plant growth of tomato plants was negatively affected by the maize microbiome, which coincided with a higher suppression of the nematodes. That raises the importance of balancing plant immunity and plant growth when managing rhizosphere microbiomes (Albrecht and Argueso, 2017; Ning et al., 2017). Prolonged effects of crop rotations on soil microbial communities are well documented (Smith et al., 2016; Ashworth et al., 2017; Benitez et al., 2017; Wubs and Bezemer, 2018). If the derived changes in microbial communities are associated with improved crop yield and yield stability, both in the presence and absence of biotic stress, then crop rotations and cover crops might be harnessed to manage soil microbiomes. This tool for sustainable agricultural intensification could be even more promising if the responsiveness of modern crops to beneficial microbiota was enhanced as a target of breeding programs. In addition, crop varieties could be selected that better support beneficial soil microbiota. However, any successful implementation in the near future requires a deeper understanding of the main taxa responsible for pathogen suppression and how different plant genotypes stimulate those taxa.

## Author Contributions

HH and JH designed the research. AE and SA performed the research. HH and AE performed the analyses. HH, JH, and AE wrote the paper.

## Conflict of Interest Statement

The authors declare that the research was conducted in the absence of any commercial or financial relationships that could be construed as a potential conflict of interest. The reviewer JEP-R and handling Editor declared their shared affiliation.
