# Supplementary material for: Rhizosphere Microbiomes Modulated by Pre-crops Assisted Plants in Defense Against Plant-Parasitic Nematodes
Source: Front Microbiol. 2018 Jun 4;9:1133. doi: 10.3389/fmicb.2018.01133 (PMC5994479; doi:10.3389/fmicb.2018.01133)
Supplement: Supplementary file 1 [file Table_1.DOCX]

**Table S1.** Root and shoot weight of soybean or tomato inoculated with different microbiomes of pre-crop rhizospheres or bulk soil, and uninoculated control. Mean and standard deviation of replicate plants are shown (n=10 for soybean, n=12 for tomato). Different letters in a column indicate significantly different means by Tukey´s test, while all plant parameters were significantly affected by the type of rhizosphere microbiome when globally tested in generalized linear models (GLM by SAS 9.4).

| Source of inoculated microbiome | Soybean plants as host | | Tomato plants as host | | | |
| --- | --- | --- | --- | --- | --- | --- |
|  | Infested by *Pratylenchus penetrans* | | Infested by *Pratylenchus penetrans* | | Infested by *Meloidogyne incognita* | |
|  | Root fresh weight (g) | Shoot dry weight (g) | Root fresh weight (g) | Shoot dry weight (g) | Root fresh weight (g) | Shoot dry weight (g) |
| Bulk soil | 3.20±0.68ab | 0.87± 0.19a | 1.60±0.25a | 0.70±0.31a | 1.65±0.21a | 0.90±0.44a |
| Soybean rhizosphere | 2.56±0.69b | 0.75± 0.26ab | 1.58±0.24a | 0.72±0.31a | 1.59±0.19a | 0.62±0.21ab |
| Maize rhizosphere | 3.32±0.56a | 0.86± 0.18a | 1.37± 0.19a | 0.41±0.17a | 1.32±0.18bc | 0.56±0.20ab |
| Tomato rhizosphere | 2.98±0.52ab | 0.77± 0.16ab | 1.63±0.15a | 0.49±0.17a | 1.59±0.19a | 0.51±0.14b |
| Uninoculated control | 2.56±0.55b | 0.60± 0.11b | 1.40±0.20a | 0.44±0.17a | 1.44±0.18ac | 0.57±0.25ab |
